# Surrogating tumour cell density in head and neck cancer: [^18^F]FDG PET- versus ADC (MRI)-based approaches

**DOI:** 10.1186/s13014-025-02716-6

**Published:** 2025-09-01

**Authors:** Athanasios Kafkaletos, Ilias Sachpazidis, Michael Mix, Montserrat Carles, Raluca Stoian, Henning Schäfer, Michael Bock, Dimos Baltas, Anca L. Grosu

**Affiliations:** 1 Division of Medical Physics, Department of Radiation Oncology, Medical Centre - University of Freiburg, Medical Faculty - University of Freiburg, German Cancer Consortium (DKTK), partner site DKTK-Freiburg, Freiburg im Breisgau, Germany; 2 Department of Nuclear Medicine, Medical Faculty, Medical Centre - University of Freiburg, University of Freiburg, German Cancer Consortium (DKTK), partner site DKTK-Freiburg, Freiburg im Breisgau, Germany; 3 Biomedical Imaging Research Group (GIBI230-PREBI) and Imaging La Fe Node at Distributed Network for Biomedical Imaging (ReDIB), Unique Scientific and Technical Infrastructures (ICTS), La Fe Health Research Institute, Valencia, Spain; 4 Department of Radiation Oncology, Medical Centre - University of Freiburg, Medical Faculty - University of Freiburg, German Cancer Consortium (DKTK), partner site DKTK-Freiburg, Freiburg im Breisgau, Germany; 5 Department of Radiology, Medical Centre - University of Freiburg, Medical Faculty - University of Freiburg, German Cancer Consortium (DKTK), partner site DKTK-Freiburg, Freiburg im Breisgau, Germany

**Keywords:** Cell density, FDG, ADC, HNSCC

## Abstract

**Objective:**

In this study we examined the correlation between standardized uptake value (SUV) of [^18^F]fluorodeoxyglucose (FDG) and apparent diffusion coefficient (ADC) within the gross tumor volume (GTV) of patients with head and neck squamous cell carcinoma (HNSCC). In addition, we assessed the comparability of cell density (*ρ*) estimates obtained from FDG PET and MRI data.

**Methods:**

Twenty-one HNSCC patients from a prospective FMISO imaging trial underwent pre-treatment PET/CT and MRI. We assessed correlations between FDG SUV (mean, max) and ADC (mean, min) within the GTV using Pearson’s correlation coefficient. The tumor cell density within the GTV was calculated from FDG SUV and from ADC maps. For the estimation of ADC-based cell density, we used a published tumor cell volume fraction (*v*_*TC*_). Agreement between FDG- and ADC-derived cell density estimates was assessed. The best-fitting *v*_TC_* was computed to achieve equal mean *ρ*_*ADC*_ and *ρ*_*FDG*_ for each patient and was compared to the literature.

**Results:**

The SUV and ADC metrics showed up to moderate negative correlations, but none of them were statistically significant at *p* < 0.05. The correlation of SUV_mean_ vs. ADC_mean_ with Pearson’s correlation coefficient *r* = −0.426 and *p* = 0.054 and SUV_max_ vs. ADC_min_ with *r* = −0.414 and *p* = 0.062 suggested a weak negative trend. The average and standard deviation of mean *ρ*_*FDG*_ and *ρ*_*ADC*_ across our cohort were (1.8 ± 0.6) × 10^8^ cells/ml and (3.3 ± 0.2) × 10^8^ cells/ml. The difference between the mean *ρ*_*FDG*_ and *ρ*_*ADC*_ was statistically significant (*p* < 0.001). To achieve equal mean *ρ*_*ADC*_ and *ρ*_*FDG*_ for each patient, the mean optimal *v*_TC_* with standard deviation was 0.29 ± 0.09. Although significantly lower than the published mean *v*_*TC*_​ (0.54), *v*_TC_* lies within the published range of *v*_*TC*_ for HNSCCs (0.28 to 0.75).

**Conclusion:**

ADC and SUV metrics exhibited moderate but marginally insignificant correlation in this dataset. Although not directly interchangeable, the two methods provide comparable, clinically relevant cell density estimates, offering flexibility to use the most accessible modality for individualized treatment planning.

**Trial registration:**

Registered at German Clinical Trials Register on 20/08/2015 (DRKS00003830).

## Background

The estimation of tumor cell density plays a critical role in advancing individualized, radiobiology-based treatment planning in radiation oncology, as tumor control probability (TCP) models rely on accurate cell density estimates to predict treatment outcomes [1–4]. Non-invasive imaging modalities such as magnetic resonance imaging (MRI) and positron emission tomography (PET) offer valuable biological information that can support tumor characterization and quantification.

Apparent diffusion coefficient (ADC) maps, derived from diffusion-weighted MRI (DWI), provide a quantitative measure of water molecule diffusion within tissues. In highly cellular tumor regions, the dense packing of cells restricts the movement of water molecules, resulting in lower ADC values. Therefore, ADC has been proposed as a surrogate marker for tumor cell density, with numerous studies reporting inverse correlations between ADC and cellularity [5–8].

On the other hand, [¹⁸F]Fluorodeoxyglucose (FDG) PET is widely used in clinical oncology to assess tumor metabolism. FDG uptake, quantified by the standardized uptake value (SUV), reflects glucose consumption by cells. Since tumor cells often exhibit elevated metabolic activity to sustain rapid proliferation, regions of higher cell density tend to show increased FDG uptake and correspondingly higher SUV values. This biological basis underpins the observed negative correlations between SUV and tumor cell density reported in several studies [9–11].

Taken together, ADC and SUV reflect different but complementary biological processes - structural cellularity and metabolic activity - that are both linked to tumor cell density. Understanding the relationship between these imaging-derived biomarkers is essential for improving the accuracy and clinical utility of quantitative imaging in radiotherapy planning.

The primary objective of this study is to assess whether cell density estimates derived from FDG PET and ADC maps are comparable. Previous research on different cancer sites has reported moderate to strong correlations between FDG SUV- and ADC-derived metrics, especially among SUV_max_, SUV_mean_, ADC_min_ and ADC_mean_ [12–16]. This study builds upon these findings by re-examining the correlation between SUV and ADC in patients with head and neck squamous cell carcinoma (HNSCC). In addition, cell density estimates obtained from FDG PET and MRI data were systematically compared to evaluate their concordance.

## Methods

### Patient cohort

Twenty one patients with HNSCC were treated with definitive chemoradiation therapy (CRT) as part of the prospective, monocentric, non-interventional FMISO imaging trial [17–19]. The FMISO trial was approved by the Independent Ethics Committee of the University of Freiburg (reference no. 479/12), German Federal Office for Radiation Protection (Bundesamt für Strahlenschutz, BfS) and the German Federal Institute for Drugs and Medical Devices (Bundesamt für Arzneimittel und Medizinprodukte, BfArM) and conducted in accordance with the Declaration of Helsinki (revised version of 2008). It is registered with the German Clinical Trials Register (DRKS00003830).

### Imaging

As part of the FMISO study, all patients underwent FDG PET/CT, planning CT and multi-parametric MRI prior to the beginning of CRT. In addition, up to three [^18^F]fluoromisonidazole (FMISO) PET/CT scans were performed to investigate tumor hypoxia: one before treatment and two during CRT, in weeks two and five. Since the current study focuses on tumor cell density estimation, only the FDG PET/CT and MRI datasets were analyzed. The median interval between those two image acquisitions was 4 days with a minimum-to-maximum range of 1 to 24 days.

We acquired the clinical FDG PET/CT images on a Philips Gemini TF BigBore 16 PET/CT scanner (Eindhoven, The Netherlands) [20, 21]. The patients were immobilised in radiation treatment position with a stereotactic face mask. Pre-therapeutic diagnostic whole-body FDG scans were performed according to the European Association of Nuclear Medicine (EANM) guidelines [22], i.e. 60 min post injection and 90 s per bed position, with a mean tracer activity of 5.0 ± 0.5 MBq/kg body weight. Subsequently, and in the same imaging session, a dedicated head and neck FDG scan was performed, with a mean duration of 9.2 ± 1.7 min and started on average 96 ± 19 min after the initial tracer injection. Image reconstruction was done iteratively on an isotropic grid size of 2 mm, using time-of-flight information and corrections for decay, dead time, normalisation, scatter and attenuation [23, 24] (BLOB-OS-TF with 3 iterations and 33 subsets). Since the standard reconstruction algorithms of the PET/CT scanner did not include explicit resolution modelling (point-spread-function modelling), an in-house-developed partial volume effect (PVE) correction algorithm was retrospectively applied to the PET images, after it was adapted for FDG PET characteristics [25]. Finally, SUV was calculated based on body weight.

We obtained the clinical MRI images on a 3 Tesla MRI system (Tim Trio, Siemens Healthineers, Erlangen, Germany) [26]. The patients were positioned on the MR system table and immobilized with MR-compatible masks. An anterior 4-element array coil was wrapped around the head-and-neck region. To maximize the local signal-to-noise ratio, the data were acquired in combination with the posterior spine coil elements within the patient table. For T2* measurements, the echo time for the multi-echo acquisition was 51 ms, the repetition time was 2510 ms, the slice thickness was 3 mm, the in-plane pixel dimensions were 2 × 2 mm² and 3 different b-values were used (b = 50, 400 and 800 s/mm²). The apparent diffusion coefficient ADC was reconstructed from the diffusion-weighted (DWI) MRI data based on the readout-segmented echo-planar-imaging technique (Resolve). The Resolve sequence used three orthogonal diffusion directions and included generalized auto-calibrating partially parallel acquisition (GRAPPA) acceleration with a factor of 2 and a partial Fourier factor of 6/8.

### Image post-processing

We imported all imaging datasets in a treatment planning system (TPS), Eclipse^®^ version 15.6 (Varian Medical Systems, Inc., Palo Alto, USA). In the present study, the primary gross tumor volume (GTV) was considered for investigation. Deformable image registrations and the contouring and propagation of the GTV were reviewed by board-certified radiation oncologists. Further details on imaging, registrations and contouring are available in [27, 28]. The CT, PET and MRI images and the corresponding structure sets were exported in DICOM format from the Eclipse TPS for further data image processing and analysis. Subsequent PVE correction, cell density estimation and statistical analyses were performed using Matlab (R2020b, Update 2, Natick, Massachusetts: The MathWorks Inc.).

### Correlation of SUV and ADC in GTV

We analyzed the correlation between SUV and ADC within the GTV, with a particular focus on ADC_min_ and SUV_max_, as they are associated with maximum cell density [5, 9]. Additionally, SUV_mean_ and ADC_mean_ were included in the analysis. To evaluate the correlation between SUV and ADC, Pearson’s correlation coefficient (*r*) was calculated along with a linear regression fit. The 95% confidence interval (CI) for each *r* was calculated with Fisher’s z-transformation estimating the precision of the correlation. We considered the correlation as none for 0 ≤ |*r*| < 2, weak for 2 ≤ |*r*| < 4, moderate for 4 ≤ |*r*| < 6, strong for 6 ≤ |*r*| < 8 and very strong for 8 ≤ |*r*| correlations. Statistically significant correlation was indicated by *p* < 0.05.

### Tumor cell density Estimation from FDG

Studies have shown that tumor cell density (*ρ*) exhibits a linear correlation with FDG uptake in vitro [9, 10]. Based on this data, the following formula can be derived to estimate *ρ* in a voxel within the GTV:

1 $$\:\rho\:=A\times\:({SUV}^{*}-1)$$

SUV* represents the standardized uptake value in a voxel, normalized to the mean SUV in a reference region of healthy tissue. In this study on head and neck tumours, the sternocleidomastoid muscle on the far side from the location of the primary tumor and the furthest from metastases was selected as such a reference region for its low and physiologically stable FDG uptake at rest and its consistent anatomical presence within the imaging field. Alternative reference regions such as blood vessels were avoided due to their susceptibility to partial volume effects, as their diameter approached the spatial resolution (full width at half maximum) of the PET system. Other blood pool regions (e.g. aortic arch) were not always fully included in the available head and neck PET/CT scans. Thus, the sternocleidomastoid muscle provided a reproducible and anatomically relevant standard for normalization in this clinical context. This normalization results in a *ρ* value of zero for voxels where SUV*=1, corresponding to areas with SUV levels similar to healthy muscle tissue.

The parameter *A*, representing the slope of the linear equation, was determined by setting the *ρ* in Eq. 1 equal to the maximum carrying capacity of tumor cells (*θ* = 10^9^ cells/ml [29]) and the SUV*, equal to the highest normalized SUV in the entire patient cohort. Since *A* depends on the choice of SUV*, we estimated the mean and the 95% CI of *A* using a bootstrap approach with 10^4^ iterations. Bootstrapping was performed by resampling the *SUV*_max_* values with replacement, recalculating the scaling parameter *A* in each iteration. The mean of this bootstrap distribution was used as the final estimate of *A*, and the 95% CI was calculated by taking the 2.5th and 97.5th percentiles of the distribution.

### Tumor cell density Estimation from ADC

We estimated the voxelized tumor cell density maps of GTVs from three-dimensional ADC maps. To exclude artefacts in ADC we applied outlier removal to the GTV voxel values using the interquartile range (IQR) method [30]. Values below Q1–1.5 × IQR were considered outliers and excluded. The factor 1.5 is derived from Tukey’s rule, which defines outliers as values significantly deviating from the central data spread. This method is robust to non-normal distributions and ensures that only statistically extreme low values, likely due to fitting errors, are removed while preserving the overall data distribution.

As demonstrated by Atuegwu et al. [5, 8], the number of cells in a voxel (N) can be estimated based on the ADC value in that voxel (*ADC*) as:

2 $$\:N=\:\theta\:\times\:\left(\frac{{ADC}_{w}\:-ADC}{{ADC}_{w}\:-\:{ADC}_{min}}\right){\times\:v}_{TC}$$

The voxel with the minimum ADC (*ADC*_*min*_) within the GTV is assumed to contain the maximum number of cells that can fit into that voxel (i.e., the carrying capacity of the voxel, *θ*) [29]. Voxels with the ADC of free water (*ADC*_*w*_ =3 × 10^−3^ mm^2^/s) are considered to have zero tumor cells. The *v*_*TC*_ is the volume fraction of the voxel that is occupied by cells, excluding the volume fractions of the extracellular extravascular space, *v*_*e*_​, and the plasma, *v*_*p*_, as described by the following equation [31]:

3 $$\:{v}_{TC}=1\:-\:{v}_{e}\:-\:{v}_{p}$$

Since the *v*_*TC*_ was not available for our cohort, we used the mean value and standard deviation of the published *v*_*TC*_ for HNSCCs out of five studies with a total of 125 cases (*v*_*TC*_ = 0.54 ± 0.23) [32–36] and used it constantly for all voxels, in all patients. The lowest and highest reported mean *v*_*TC*_ were 0.28 and 0.75 respectively. Finally, *ρ*_*ADC*_ was calculated by dividing *N* by the voxel volume.

### Comparison of tumour cell density estimates from FDG and ADC

To assess the potential interchangeability of FDG- and ADC-based approaches, we evaluated the statistical significance of the differences between the mean *ρ*_*FDG*_ and *ρ*_*ADC*_ within the GTV, for each of the 21 patients. We propagated the standard error (SE) of *A* to the mean *ρ*_*FDG*_ and the SE from the published *v*_*TC*_ to the mean *ρ*_*ADC*_.

For the mean *ρ*_*FDG*_ in the GTV for each patient, the propagation of SE was.

4 $$S{E_{\rho {\:_{FDG,mean}}}}\\= \sqrt {\left( {(SU{V_{mean}} - 1} \right) \times \:S{E_A}{)^2} + {{(A \times \:S{E_{SU{V_{mean}}}})}^2}}$$

where SUV_mean_ and SE_SUVmean_ are the mean and SE of the SUV values within the GTV. For the mean *ρ*_*ADC*_ in the GTV for each patient, the propagation of SE was.

5 $$S{E_{\rho {\:_{ADC,mean}}}}\\= \left| {\theta \: \times \:\left( {\frac{{AD{C_w}\: - ADC}}{{AD{C_w}\: - \:AD{C_{min}}}}} \right)} \right| \times \:S{E_{{v_{TC}}}}$$

These patient-specific SEs were then incorporated into a non-parametric bootstrap analysis with 10^4^ iterations to test the difference between the two modalities. In each iteration, we resampled the 21 patients with replacement, added Gaussian noise to the resampled *ρ*_*FDG*_ and *ρ*_*ADC*_, based on their propagated SEs, and calculated the mean difference between the two sets. The 95% CI of the mean difference was obtained from the 2.5th and 97.5th percentiles of the resulting distribution.

Finally, to assess the consistency between *ρ*_*FDG*_ and *ρ*_*ADC*_, we calculated the optimal *v*_TC_*, for each case by equating *ρ*_*ADC*_ to *ρ*_*FDG*_ (Eq. 6).

6 $$\rho {\:_{FDG}}\\= \theta \: \times \:\left( {\frac{{AD{C_w}\: - ADC}}{{AD{C_w}\: - \:AD{C_{min}}}}} \right) \times \:{v_{TC}}^*$$

The published *v*_*TC*_ served as a reference for comparison. Additionally, a 95% CI was computed for the mean of *v*_TC_* to assess the range within which the true mean is expected to lie. An overlap of the 95% CI of *v*_TC_* and the published *v*_*TC*_ range of 0.28 to 0.75 was assessed.

## Results

### Correlation of SUV and ADC in GTV

**Fig. 1:**
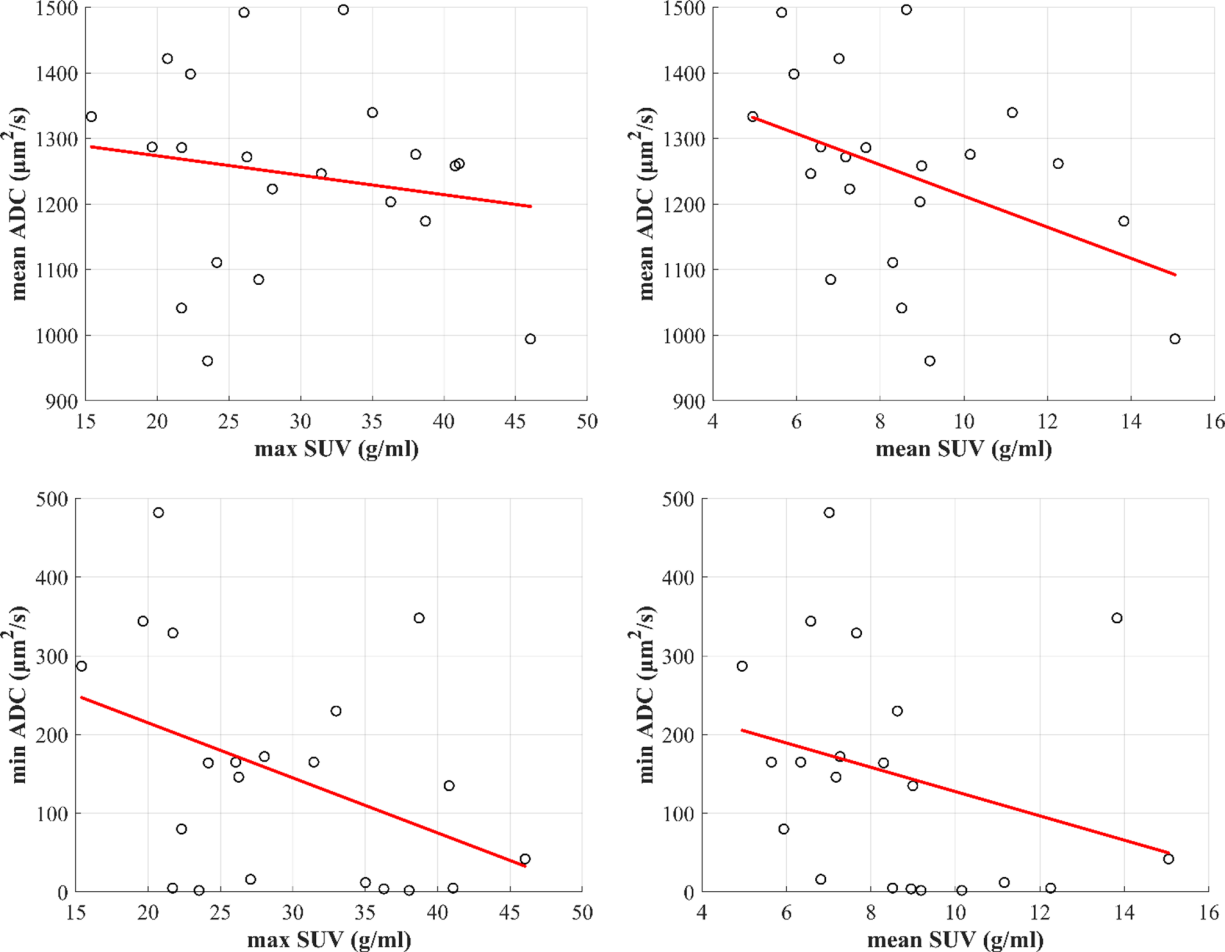
Linear regression lines for ADC and SUV values are shown for each metric combination across the 21 cases

Pearson correlation analysis revealed low to moderate negative correlations between SUV_max_ and ADC_min_ as well as SUV_mean_ and ADC_mean_. However, none of these correlations were statistically significant (*p* ≥ 0.05). The results of the analysis are summarized in Table 1. As shown in Table 1, the negative correlation between SUV_mean_ and ADC_mean_ was marginally insignificant (*p* = 0.054). The scatter plots of the SUV and ADC values, along with fitted regression lines are presented in Fig. 1.

**Table 1:** The correlation analysis and linear regression results including the pearson’s correlation coefficient (*r*) with its 95% CI and the *p* value that demonstrates the significance of the correlation.

Metrics		*r*	95% CI(*r*)		*p*	Slope	Intercept
SUV_max_	ADC_min_	−0.414	(−0.718,	0.022)	0.062	−6.99	355
SUV_max_	ADC_mean_	−0.169	(−0.560,	0.283)	0.463	−2.96	1333
SUV_mean_	ADC_min_	−0.287	(−0.640,	0.165)	0.207	−15.43	282
SUV_mean_	ADC_mean_	−0.426	(−0.725,	0.007)	0.054	−23.74	1450

### Comparison of tumour cell density estimates from FDG and ADC

**Fig. 2:**
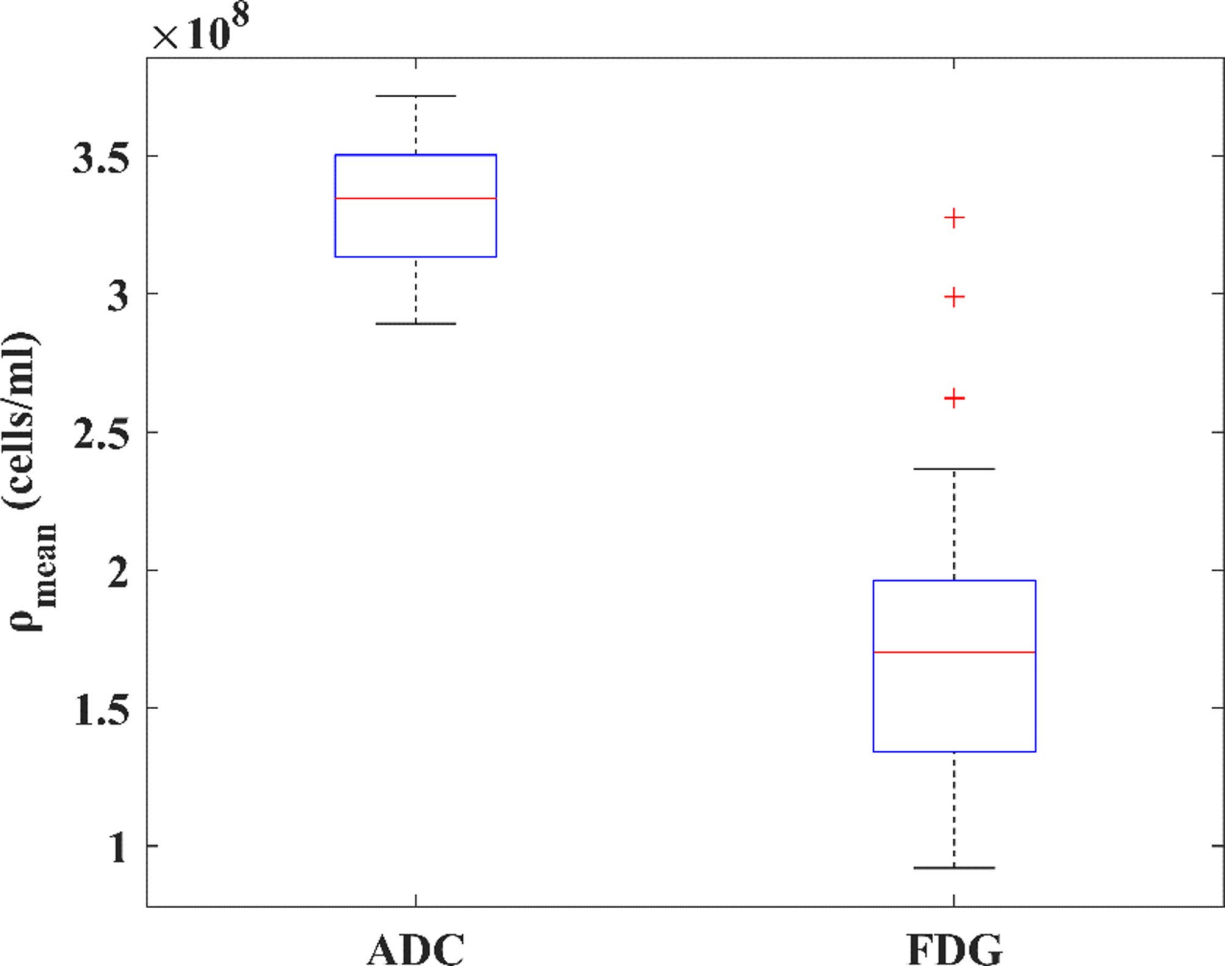
The boxplots of the mean *ρ*_*ADC*_ and *ρ*_*FDG*_ for the 21 cases. On each box, the central mark indicates the median, and the bottom and top edges of the box indicate the 25th and 75th percentiles, respectively. The whiskers extend to the most extreme data points not considered outliers, and the outliers are plotted individually using the ‘+’ marker symbol

The estimated mean of *A* from the bootstrapping method was 2.3 × 10^7^ cells/g and its 95% CI was 2.2 × 10^7^ cells/g to 2.7 × 10^7^ cells/g. The average and standard deviation of the observed mean and maximum *ρ*_*FDG*_ in our cohort were (1.8 ± 0.6) × 10^8^ cells/ml and (6.6 ± 1.9) × 10^8^ cells/ml respectively. The mean published *v*_*TC*_ and its standard deviation (*v*_*TC*_ = 0.54 ± 0.23) from five studies on a sum of 125 HNSCC cases was used to calculate the mean *ρ*_*ADC*_ according to Eq. 2. The mean *ρ*_*ADC*_ with its standard deviation was (3.3 ± 0.2) × 10^8^ cells/ml on average. The boxplots of the mean values of *ρ*_*FDG*_ and *ρ*_*ADC*_ across our cohort are shown in Fig. 2.

A mean difference of 1.56 × 10^8^ (95% CI: 1.30 × 10^8^ to 1.81 × 10^8^) between the mean *ρ*_*FDG*_ and *ρ*_*ADC*_ was demonstrated, taking into account the corresponding SE. The statistical analysis indicated a significant difference between the two approaches (*p* < 0.001). The mean *v*_TC_* over 21 cases was 0.29 (range: 0.15 to 0.48) fell within the reported range of values in published studies (0.28 to 0.75). Also the higher end of the range for the mean *v*_TC_* overlaps with the low end of the reported range for *v*_*TC*_.

## Discussion

In this study, we investigated the comparability of cell density estimates derived from FDG PET and ADC maps in patients with HNSCC. Expanding on previous findings that reported correlations between SUV- and ADC-derived metrics, we re-examined the relationship between these imaging parameters. Beyond the correlation analysis, we estimated the tumor cell density from both imaging modalities and systematically compared the results.

No statistically significant correlation was observed between SUV and ADC metrics. The strongest but still not significant relationship appeared between SUV_mean_ and ADC_mean_ with Pearson’s correlation coefficient *r* = −0.426 and *p* = 0.054 and for SUV_max_ vs. ADC_min_ with *r* = −0.414 and *p* = 0.062, suggesting a moderate negative trend in both combinations. A meta-analysis of the correlation between ADC and FDG SUV for multiple cancer sites by Deng et al. was published in 2017 [16]. The analysis was based on 49 studies with a total of 1927 patients and for combined correlation coefficients between ADC and SUV reported a pooled *r* of −0.35 with a 95% CI from −0.42 to −0.28 among all included studies. There was also significant heterogeneity with an inconsistency index *I*^2^ = 78.4%. Higher correlations were found in the brain tumor, cervix carcinoma and pancreatic cancer. For HNSCCs in particular, the reported combined *r* from 6 studies was −0.31 (95% CI: –0.44, –0.19) and the heterogeneity was not significant at *I*^2^ = 11.0%.

The high heterogeneity observed in the overall analysis reflects the variability in tumor biology, imaging protocols, and patient populations across different cancer sites. In contrast, the low heterogeneity among head and neck studies, in combination with the fact that 4 out of 6 studies did not show significant correlation of the ADC and SUV metrics in HNSCCs, indicates a consistently low correlation for HNSCCs. This absence of statistically significant correlations between ADC and SUV metrics, also observed in our analysis, may reflect the complex tumor biology of HNSCC. In particular, local factors such as necrosis, inflammation, and heterogeneous stromal composition can influence FDG uptake and water diffusion differently. For instance, inflammatory cell infiltration can lead to increased FDG uptake without necessarily altering tissue cellularity. In contrast, necrotic regions are characterized by increased membrane permeability and cell membrane breakdown, resulting in less restricted water diffusion and therefore higher ADC values [37]. These areas may simultaneously exhibit low FDG uptake due to lack of viable, metabolically active cells. Such spatial mismatches and opposing effects could contribute to the observed variability and weaken the correlation between metabolism- and diffusion-based imaging biomarkers.

Regarding cell density, for the mean *ρ*_*ADC*_ and *ρ*_*FDG*_ to be equal for each patient, the optimal *v*_TC_* was on average 0.29. According to previous studies, *v*_*e*_​ values range from 0.20 to 0.70 and the values for *v*_*p*_​ range from 0.05 to 0.09, leading to a range for *v*_*TC*_​ from 0.28 to 0.75 [32–36]. Although the mean *v*_TC_* in our data was significantly lower than the published mean value for *v*_*TC*_, *v*_TC_* lies within the published range of *v*_*TC*_ for HNSCCs. This indicates that despite the lack of direct interchangeability, the two methods yield comparable estimates within the same order of magnitude, supporting the potential use of either method in relevant contexts.

Despite the comparable order of magnitude between methods, the estimated cell densities differed substantially, with an average difference of about 60%. This difference is based on a fixed *v*_*TC*_ value from the literature; individualized *v*_*TC*_ values could potentially reduce the discrepancy, but substantial differences may still remain. Such a difference in estimated cell density (FDG vs. ADC), for a treatment course of total prescription dose of 70 Gy, translates into only a ~3% increase in the equivalent dose in 2 Gy fractions required to achieve the same tumor control probability. This relatively small impact on the required dose suggests that both imaging modalities, despite differences in absolute cell density estimation, can offer comparable guidance for treatment planning. However, if one modality consistently over- or underestimates cell density across patients or tumor types, it could lead to systematic over- or underestimation of the required dose. Therefore, cross-validation between modalities may improve confidence in tumor burden estimation and help tailor dose painting strategies. When both modalities agree, this supports the robustness of the estimated cell density and strengthens the basis for personalized dose prescription.

The use of a fixed volume fraction of tumor cells from the literature is a limitation, as *v*_*TC*_ varies between patients and tumor regions. A fixed *v*_*TC*_ value may introduce inaccuracies in cell density estimation, potentially affecting the precision and personalization of treatment planning based on ADC-derived metrics. On the contrary, individual *v*_*TC*_ estimation requires dynamic contrast-enhanced (DCE) MRI, which provides voxelized maps of *v*_*p*_ and *v*_*e*_ fractions. These measurements add technical complexity, longer scanning times, and may reduce patient compliance, limiting feasibility in clinical practice.

Furthermore, there is a lack of formal assessment of inter-observer variability in GTV delineation in this study. However, a standardized protocol was followed where GTVs were initially defined using a 40% SUV_max_ threshold on [¹⁸F]FDG PET and subsequently refined and evaluated by experienced radiation oncologists based on co-registered CT and MRI data, in line with the clinical study protocol [17, 19]. While some variability due to observer input or image registration cannot be entirely ruled out, the multi-modality and multi-step delineation approach was designed to enhance consistency and reduce subjectivity. Re-contouring or multiple observer comparisons were not feasible retrospectively. While minimum ADC and maximum SUV values are expected to lie well within the GTV and thus be less affected by contour variability, mean metrics may be influenced.

In our analysis, the relatively small cohort of 21 patients presents several limitations that may have affected the results. The limited sample size reduces statistical power, making it more difficult to detect true associations between imaging biomarkers and cell density. As a result, observed correlations may not reach statistical significance, even if underlying relationships exist. Additionally, small cohorts are more susceptible to the influence of outliers and individual variability, which can distort correlation estimates and reduce the stability and reliability of the findings. These limitations also affect the generalizability of our results, as a small sample may not fully represent the broader patient population. To address these issues, future studies should aim to include larger and more diverse patient groups, which would help validate the findings, reduce the influence of variability, and improve confidence in the robustness of the proposed imaging-based cell density estimation methods.

Another key limitation of the study is the variable time interval between FDG-PET and MRI acquisitions, which could affect the comparability of the two imaging modalities. Although the median interval was relatively short (4 days), 14 out of 21 patients had an interval of less than 5 days and 17 had an interval of less than 10 days. However, in 4 cases the scans were performed 11 to 24 days apart, introducing a potential risk of biological changes, such as proliferation, necrosis, or inflammation, that could alter both FDG uptake and diffusion characteristics. Such temporal discrepancies may reduce the reliability of comparisons between cell density estimates derived from the two modalities.

Beyond the study-specific limitations, it is also important to consider the inherent limitations of each imaging-based approach which can influence the accuracy of SUV- and ADC-based cell density estimates. The linear correlation of FDG uptake and cell count has been established in vitro, therefore FDG uptake can be used to estimate cell density in vivo assuming that certain conditions apply. Glucose metabolism should be the dominant driver of FDG uptake, minimizing confounding factors such as inflammation, hypoxia, or necrosis, which can alter glycolytic activity independently of cell count. Each viable cell should also take up a similar amount of FDG; however, this assumption may be compromised in vivo due to perfusion heterogeneity, as FDG delivery depends on regional blood flow. Importantly, in vivo conditions are more complex, and factors such as tumor microenvironment heterogeneity, variable cellular metabolism, and partial volume effects may influence FDG uptake independently of cell density. Therefore, while the in vitro linear relationship provides a useful model, caution is warranted when applying it clinically, and further validation with in vivo studies is necessary to confirm its robustness under physiological conditions. Additionally in the FMISO imaging trial, the GTV was delineated using information from the FDG SUV plus multi-parametric MRI, potentially adding regions to the GTV with lower SUV and therefore, decreasing the calculated mean SUV and mean estimated *ρ.*

ADC is more directly related to cell density since it reflects water diffusion restriction in highly cellular areas and is not affected by metabolic variations like FDG uptake. However, ADC might be affected by image artefacts and reconstruction errors, particularly in regions such as the neck where magnetic field inhomogeneities are present. This clearly influences the accuracy of the correlation between ADC and SUV metrics. Therefore, we applied lower outlier removal based on the IQR. The percentage of excluded voxels was low overall (median: 0.07%, range: 0.00 to 1.26%), with no outliers found in 9 out of 21 cases. While this filtering step had minimal impact on mean ADC values, it was particularly important for ensuring reliable minimum ADC estimates by excluding near-zero values that often arise from artefacts or failed fits.

For clinical implementation, FDG-based estimation can be more feasible, as PET is already integrated into treatment workflows, and SUV-based calculations can be automated. On the other hand, ADC-based estimation may be more biologically robust but requires standardization, making it less ideal for routine clinical use. The calculation of *N* using Eq. 2 requires both DWI and dynamic contrast-enhanced (DCE) MRI for *v*_*TC*_ estimation. As a result, ADC-based cell density calculation for patients is more complex and time-consuming. Nevertheless, while the two methods are not directly interchangeable, the fact that they yield comparable cell density estimates within the same order of magnitude suggests that both may provide clinically relevant information. Depending on the clinical demands for pre-treatment molecular functional imaging, the acquisition of multiple MRI sequences may be more advantageous than dual PET scans (FMISO and FDG) on different days and vice versa. Having two complementary methods for estimating cell density broadens the tools available for biological tumor characterization and may support individualized treatment strategies when one modality is not available or feasible.

## Conclusion

In summary, ADC and SUV metrics do not exhibit a strong correlation in our cohort, highlighting the complexity of using these imaging modalities interchangeably for cell density estimation. While systematic differences are present, the resulting cell density values remain comparable, supporting the potential use of either method in relevant contexts and increasing flexibility in clinical settings. Future studies incorporating individual *v*_*TC*_ measurements could further refine the multi-parametric MRI-based tumor cell density estimation and enable a more accurate comparison to the FDG-based method.

## Data Availability

No datasets were generated or analysed during the current study.

## Abbreviations

ADC: Apparent Diffusion Coefficient
CI: Confidence Interval
CRT: Chemoradiation Therapy
DCE: Dynamic Contrast-Enhanced
DWI: Diffusion-Weighted MRI
EANM: European Association of Nuclear Medicine
FDG: [^18^F]Fluorodeoxyglucose
GRAPPA: generalized auto-calibrating partially parallel acquisition
GTV: Gross Tumor Volume
HNSCC: Head and Neck Squamous Cell Carcinoma
IQR: Interquartile Range
MRI: Magnetic Resonance Imaging
PET: Positron Emission Tomography
PVE: Partial Volume Effect
SE: Standard Error
SUV: Standardized Uptake Values
TCP: Tumor Control Probability
TPS: Treatment Planning System
